# Deficits in Monocyte Function in Age Related Macular Degeneration: A Novel Systemic Change Associated With the Disease

**DOI:** 10.3389/fmed.2021.634177

**Published:** 2021-03-17

**Authors:** Ben J. Gu, Xin Huang, Pavan K. Avula, Emily Caruso, Candace Drysdale, Kirstan A. Vessey, Amber Ou, Christopher Fowler, Tian-Hua Liu, Yong Lin, Adam Horton, Colin L. Masters, James S. Wiley, Robyn H. Guymer, Erica L. Fletcher

**Affiliations:** ^1^The Florey Institute of Neuroscience and Mental Health, The University of Melbourne, Parkville, VIC, Australia; ^2^National Clinical Research Center for Aging and Medicine, Huashan Hospital, Fudan University, Shanghai, China; ^3^Department of Surgery (Ophthalmology), Centre for Eye Research Australia, Royal Victorian Eye and Ear Hospital, University of Melbourne, East Melbourne, VIC, Australia; ^4^Department of Anatomy and Neuroscience, The University of Melbourne, Melbourne, VIC, Australia

**Keywords:** monocytes, phagocytosis, drusen, reticular pseudodrusen, glatiramer acetate

## Abstract

Age-related macular degeneration (AMD) is characterized by the accumulation of debris in the posterior eye. In this study we evaluated peripheral blood monocyte phagocytic function at various stages of AMD and in aged matched control participants. Real-time tri-color flow cytometry was used to quantify phagocytic function of peripheral blood monocyte subsets (non-classic, intermediate and classic) isolated from subjects with intermediate or late AMD and compared with age matched healthy controls. Assessment of phagocytic function of monocytes isolated from those with and without reticular pseudodrusen was also made, and the effect of glatiramer acetate on phagocytic function assessed. Phagocytic function was reduced in all subjects with AMD, irrespective of stage of disease. However, there was no correlation between phagocytic function and drusen load, nor any difference between the level of phagocytosis in those with or without reticular pseudodrusen. Treatment with glatiramer acetate increased phagocytosis of classical and non-classical monocytes, normalizing the reduction in phagocytosis observed in those with AMD. These findings suggest that defective systemic phagocytosis is associated with both intermediate and late stages of AMD, highlighting a potential role in the accumulation of debris that occurs early in the disease process. Assessing peripheral monocyte phagocytic function provides further insights into the etiology of this disease and offer a novel therapeutic target.

## Introduction

Age related macular degeneration (AMD) is the leading cause of irreversible vision loss in the western world (1). The early stages of the disease develop in ~1 in 7 people over the age of fifty, of whom ~1 in 7 progress to vision threatening late AMD (2). Drusen, the early clinical sign of AMD, are lipid rich deposits located beneath the retinal pigment epithelium (RPE) whose increasing size is associated with increasing risk of progression to vision threatening late disease; characterized by atrophic changes in the retina called geographic atrophy or aberrant vascular pathology called choroidal neovascularization (3, 4). Recently, another distinct deposit seen clinically as reticular pseudodrusen (RPD), located between the RPE and photoreceptors, as sub retinal drusenoid deposits, has emerged and their importance highlighted in disease progression (5–7). These different deposits, in combination with an accumulation of lipid rich deposits within Bruch's membrane (BM), a semi-permeable membrane located between the choroidal blood supply and the retina, are considered integral to the pathogenesis of AMD. The underlying mechanisms that lead to drusen and RPD formation and accumulation of debris in BM, however, are poorly understood.

Inflammation and innate immunity are thought to play a role in the pathogenesis of AMD with complement activation, retinal microglial activation and choroidal macrophage infiltration being key components (8–11). Genetic mutations in the alternative complement pathway, particularly involving the complement factor H (CFH) gene, are associated with an increased risk of development of AMD (12–14) and infiltrating mononuclear phagocytes have been identified within drusen as well as lesions associated with more late disease (15). One of the central functions of innate immunity involves tagging of pathogens or extracellular debris for binding, engulfment and removal by immune cells (16). While much data has established a major role of the RPE in phagocytosis of products generated from the turnover of photoreceptor outer segments, mononuclear phagocytes including monocytes and retinal microglia in the subretinal space also perform a phagocytic function and express scavenger receptors including CD36 (17), MER receptor tyrosine kinase, CX3CR1 (10) as well as integrins such as CD11b and CD11c (18).

Previous studies indicate that circulating monocytes are altered in those with late AMD (19, 20). In particular, gene expressional studies demonstrate broad changes in specific subtypes of monocytes in those with neovascular AMD (19), and differences in transcriptome between the distinct forms of late AMD (20). Our previous work has implicated defects in monocyte phagocytosis in late AMD (21, 22). P2X7 receptors are expressed by peripheral blood monocytes/macrophages, as well as retinal microglia, where they act as a scavenger receptor of non-opsonized beads, live and dead bacteria, as well as apoptotic cells (23–25). Inheritance of a rare haplotype of P2X7 G150R and P2X4 Y315C is associated with increased risk of late AMD as well as reduced phagocytosis (21). In addition, P2X7null mice show reduced phagocytic rate in monocytes and retinal microglia in association with signs of early stages of AMD including thickening of Bruch's membrane (22).

Here, we hypothesized that dysregulation of monocyte phagocytosis may occur in those with AMD and in particular, is present in the early stage of the disease, when debris accumulates in several locations within the posterior eye in the form of drusen, RPD and deposits within BM. We, therefore, sought to assess the peripheral pool of monocytes for their phagocytic ability at different stages of AMD. In addition, we and others have shown that defective phagocytosis can be reversed, in part, by glatiramer acetate, a currently available treatment of multiple sclerosis, which acts on monocyte to facilitate phagocytosis (26–28). Therefore, in this study, we also examined the *in vitro* effect of glatiramer acetate on phagocytic function in cells isolated from participants with AMD.

## Materials and Methods

### Human Subjects

Participants (all Caucasians) with AMD were recruited from Center for Eye Research Australia (CERA, East Melbourne, Australia). The inclusion criteria for all AMD participants included being 50 years of age or older and for the early stages of AMD they had drusen ≥125 μm in both eyes [Beckman intermediate stage of AMD (4)] and no late AMD (4). We refer to these subjects as “intermediate” AMD (iAMD). Participation in a sub threshold laser intervention study (29), was permitted as long as participants had not received laser treatment for at least 6 months at the time of blood draw. For participants with late-stage AMD, at least one eye had to have geographic atrophy (GA) or choroidal neovascularization (CNV). Cases undergoing treatment for CNV were included only if their last treatment was >30 days prior to blood draw.

Healthy controls (HC) aged 50 or over, and all Caucasians were recruited either through CERA or through the Australian Imaging Biomarker and Lifestyle study of aging (AIBL), usually as friends or unrelated relatives of cases. Controls were all assessed in an identical manner as the AMD cases and graded as having no drusen > 63 μm in either eye (4). Exclusion criteria for both cases and controls included other ocular diseases that could compromise the ability to examine the retina and any medication known to affect retinal health. Participants with diabetes, uncontrolled hypertension, neurological or systemic disease affecting vision, or had systemic inflammatory disease, or were on treatment with anti-inflammatory medication were also excluded. All cases and controls had their visual acuity tested, had multimodal imaging of the retina and a clinical ocular examination. Written informed consent was obtained from all participants and the research adhered with the Declaration of Helsinki.

### Ocular Imaging

In order to quantify the level of drusen burden, multimodal imaging was performed that included color fundus photography (CFP; Canon CR6-45NM; Canon, Saitama, Japan), near-infrared reflectance (NIR) and fundus autofluorescence (FAF) using 488 nm blue light excitation, and SD-OCT scans using a Spectralis HRA+OCT device (Heidelberg Engineering, Heidelberg, Germany) and Cirrus Zeiss SD-OCT (Carl Zeiss Meditec, Dublin, CA). The grading of the color fundus images was performed using OptomizePro (Digital Healthcare Image Management System, Digital Healthcare Ltd., Cambridge, UK) by experienced graders to determine the Beckman stage of AMD (4). The drusen area and volume within a 3- and 5-mm diameter circle centered on the fovea was automatically calculated from the Cirrus OCT. A close correlation was found between drusen area and volume and thus total drusen area within a 5 mm circle centered on the fovea in both eyes was used as the measure of drusen load.

The presence of absence of reticular pseudodrusen in those with intermediate AMD was confirmed based on multimodal imaging using OCT as well as imaging with NIR, FAF or CFP. The total RPD area, as a percent of the total fundus area, was measured on both the NIR and fundus FAF images using image J software to determine the area occupied by RPD. The greater of the two measurements was taken as the area of RPD. The graders were masked to the results of the phagocytosis assays.

#### Leucocyte Phenotyping

Human peripheral blood was collected into EDTA anti-coagulant Vacutainer® tubes. For surface staining, aliquots of 100 μL fresh blood were added into FACS® tubes with pre-mixed antibody cocktails. Fluorescein isothiocyanate (FITC) conjugated anti-human CD16 and CD19 monoclonal antibody (mAb) were from DAKO; FICT-conjugated anti-CD15 mAb, R-Phycoerythrin (R-PE) conjugated anti-CD16, Peridinin chlorophyll (PerCP) conjugated anti-human CD14, Allophycocyanin (APC) conjugated anti-human CD14 and CD3 R-phycoerythrin (RPE) conjugated anti-human CD11b, anti-CD33 mAb, FICT-conjugated anti-CD34 mAb, Peridinin chlorophyll (PerCP) conjugated anti-human CD14, Allophycocyanin (APC) conjugated anti-human CD14 and CD11c mAb were from BD Bioscience (New Jersey, USA). The titration of each antibody was determined by an initial saturation test. The mixture was incubated for 15 min at room temperature with gentle shake, followed by the addition of 2 mL of BD FACS Lysing solution (BD Biosciences Cat# 555899). After 15 min, equal volume of phosphate buffered saline (PBS) was added and leukocytes were centrifuged at 1,400 rpm for 4 min. The cells were then resuspended into 0.3 mL PBS. The cells were kept at 4°C in the dark and analyzed on the same day using a FACSCalibur^TM^ flow cytometer. The neutrophils, lymphocytes and monocytes populations (counts and percentage) were initially gated according to their forward and side scatters, then further gated with specific CD markers (CD15 and/or CD16 for neutrophils, CD14 and CD16 for monocytes, CD3 for T lymphocytes and CD19 for B lymphocytes). Results were analyzed using Flowjo software (version 7.65&10).

### Real-Time *in vitro* Phagocytosis of Beads Assay

Monocyte phagocytosis was performed as previously described (28). Briefly, human peripheral blood mononuclear cells (PBMCs) (2 × 10^6^/mL) were labeled with APC-CD14 and FITC-CD16 mAb, the two main cell surface markers that distinguishes human monocyte subsets: classical (CD14^+^CD16^−^); intermediate (CD14^+^CD16^+^) and non-classical (CD14^dim^CD16^+^) (30). After washing, cells were incubated in 5 μL of 1.0 μm Fluoresbrite^Ⓡ^ carboxylate 1.0 μm Yellow-Orange (YO) latex microspheres (Polysciences, Warrington, PA, USA) and the level of uptake of fluorescent beads quantified over 6 min using a FACSCalibur flow cytometer (BD Bioscience) with TimeZero attachment to maintain cuvette temperature at 37°C. The area under the bead uptake curve in the first 6 min was used to calculate phagocytosis using Excel (Microsoft) (31). In some experiments, 100 μg/mL glatiramer acetate (Copaxone®, 20 mg/mL; TEVA Pharmaceutical Industries Ltd., Petah Tikva, Israel) was applied to cells for 10 min at 37°C prior to performing the bead uptake assay.

### Statistical Analysis

Results are expressed as mean ± standard deviation. One- or Two-way ANOVA was used where comparisons across two factors or more means were evaluated and a Tukeys *post-hoc* test was used to make individual comparisons as appropriate (GraphPad Prism version 9; GraphPad Software, Inc.). In order to account for age, gender or previous laser treatment, analysis of covariance (ANCOVA) was performed using SPSS25 (IBM). Data presented in all graphs is unadjusted for age, gender or laser and, with *p*-values in the graphs relate to unadjusted comparisons.

## Results

A total of 104 participants with AMD; 72 with intermediate AMD (iAMD) (mean age: 69.4 ± 7.7 years) and 32 with late AMD (mean age 77.8 ± 6.9) as well as 74 healthy aged-matched control subjects (mean age 73.0 ± 6.9) were investigated (Table 1). As a consequence of the amount of sample available for each subject, it was not possible to undertake all experiments on each subject. Therefore, some subjects were assessed for monocyte phagocytosis whilst others were used for expression studies using flow cytometry, as indicated.

**Table 1:** Summary of participants in this study.

**Group**	**Total**	**Sex (female/male)**	**Age**	***P* (vs. HC)**
Healthy controls (HC)	74	44/30	73.0 ± 6.9	-
AMD (all)	104	70/34	72.0 ± 8.4	0.3687
Intermediate^#^	72	53/19	69.4 ± 7.7	0.0031
Late AMD^*^	32	17/15	77.8 ± 6.9	0.0018

# *Includes 42 with no RPD, 18 with RPD*.

* *Include 17 GA and 15 CNV patients*.

### Monocyte Phagocytosis Is Reduced in Intermediate AMD

**Figure 1:**
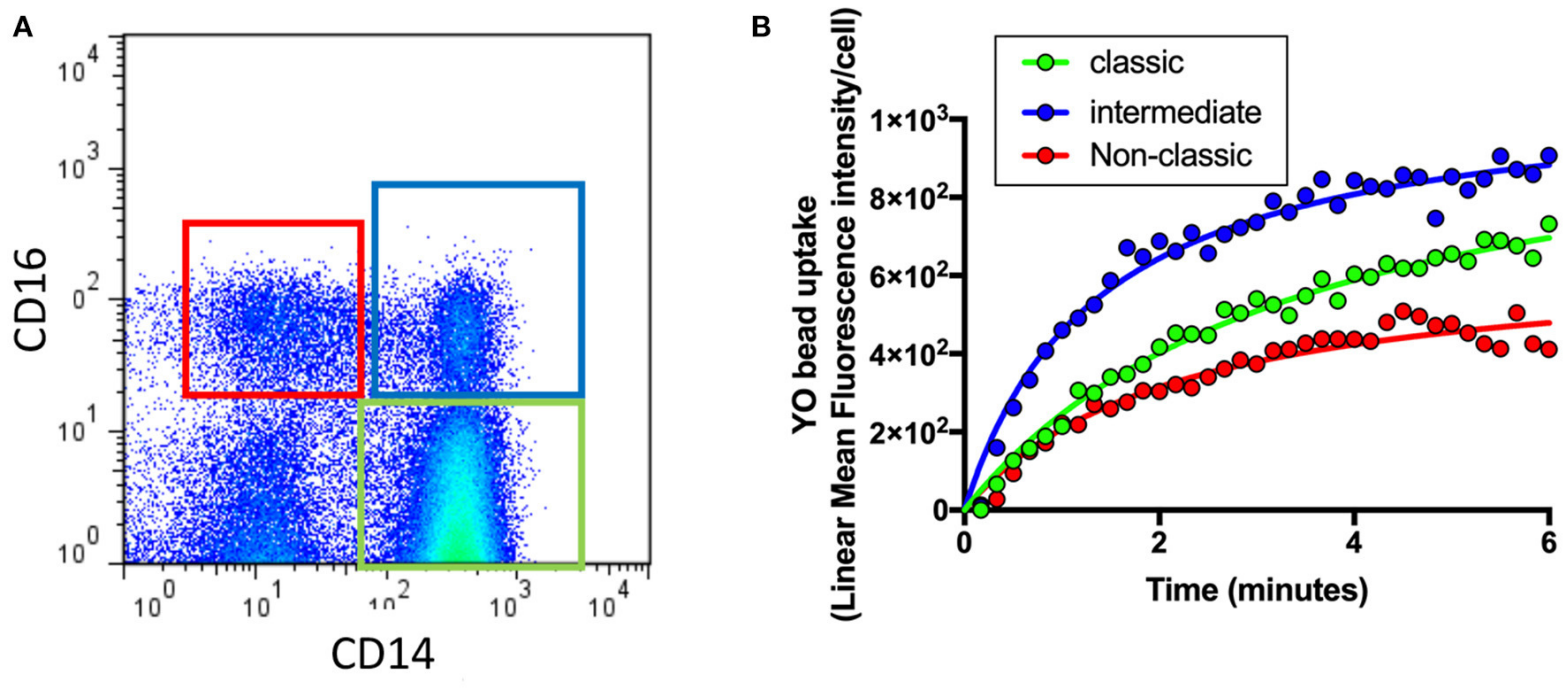
Quantification of phagocytosis across the different types of monocytes. Whole blood leukocytes were labeled with different surface markers in four-color flow cytometry panels. **(A)** A typical gate strategy used in YO bead uptake for gating CD14^dim^CD16^+^ nonclassical monocytes (red), CD14^+^CD16^+^ intermediate monocytes (blue) and CD14^+^CD16^−^ classic monocytes (green). **(B)** The corresponding kinetic curve of YO beads uptake in gated populations are shown.

As shown in Figure 1A, flow cytometry was used to identify different monocyte populations based on their expression of CD14 and CD16. Phagocytosis of each cell class was then evaluated by quantifying uptake of fluorescent beads (YO beads) over time in those with intermediate AMD (*n* = 60), advanced AMD (*n* = 30) and healthy controls (*n* = 35). In a typical healthy control subject, intermediate monocytes (CD14^+^CD6^+^) showed the highest level of phagocytosis of all three types of monocytes, whilst. classic monocytes (CD14^+^CD16^−^) displayed lower levels of phagocytosis and non-classic monocytes (CD14^−^CD16^+^) showed an even lowest level of phagocytosis (Figure 1B).

**Figure 2:**
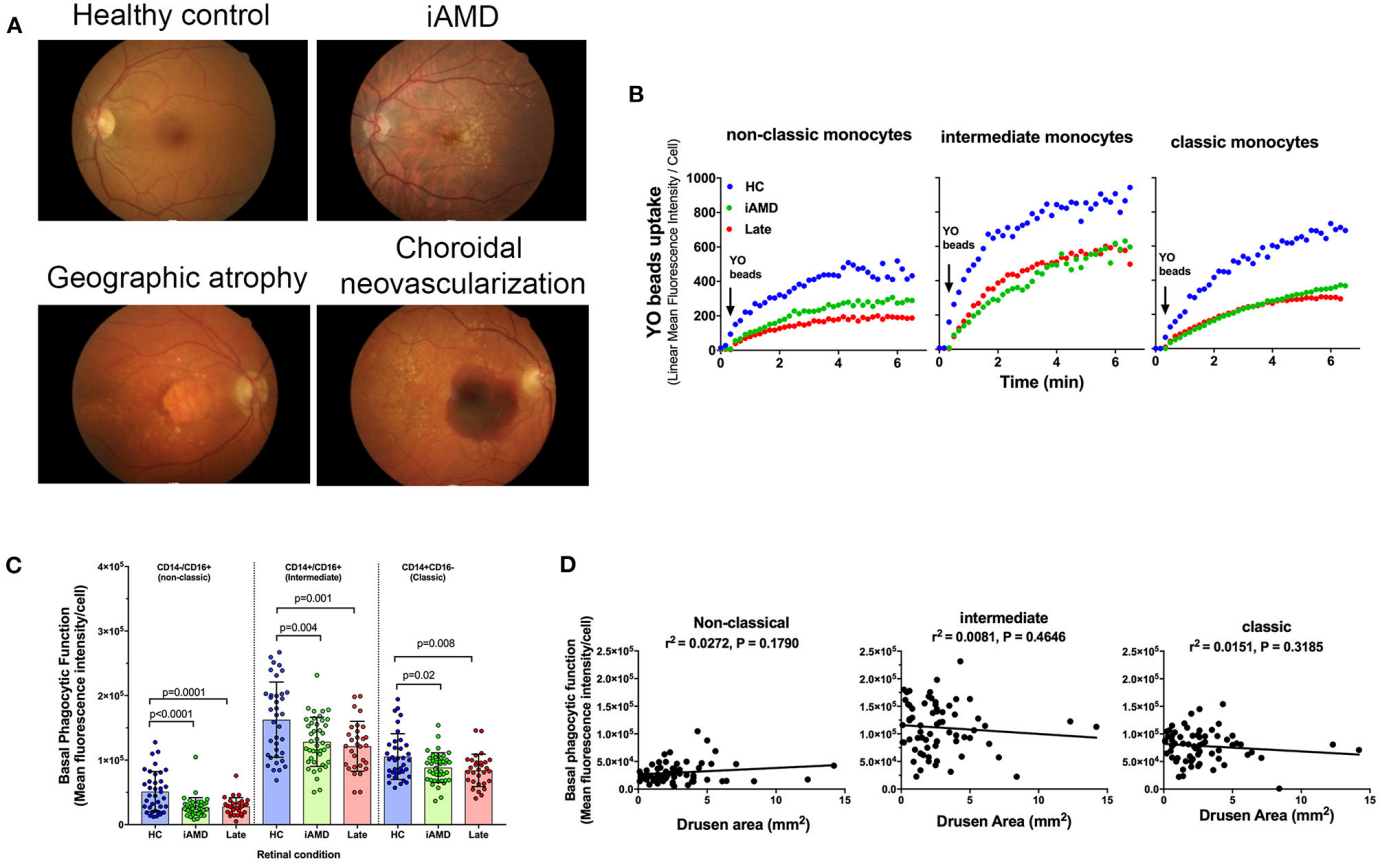
Monocyte phagocytosis in subjects with AMD. **(A)** Representative fundus images from a healthy control (normal) subject, person with intermediate AMD (iAMD), geographic atrophy and choroidal neovascularization. **(B)** A typical example of YO beads uptake curve by three monocyte subsets from a healthy control (HC; blue) a patient with intermediate AMD (green) and a subject with late AMD (red) Fresh human peripheral blood monocytes (PBMCs) were labeled with APC-conjugated CD14 and FITC-conjugated CD16 before the addition of 1 μm YO beads. The YO beads fluorescence intensity was analyzed by real time flow cytometry. **(C)** Graph showing mean ± standard deviation basal phagocytic function of monocytes subsets isolated from healthy control subjects (HC) (*n* = 35), subjects with iAMD (iAMD) (*n* = 61) and subjects with late AMD (*n* = 30). *P*-values from One-way ANOVA analysis and Tukey's multiple comparisons tests are shown for comparison between specific groups. **(D)** Correlations of basal phagocytic function for the three monocyte subtypes compared to drusen area. There was no significant correlation between phagocytosis and drusen area for any of the monocytes examined.

Phagocytic function of monocytes isolated from healthy control subjects, those with intermediate and those with advanced AMD was assessed. Representative fundus images of each participant type are shown in Figure 2A. Figure 2B shows representative uptake curves for each of the three monocyte subtypes that were isolated from a healthy control (blue circles), a subject with intermediate AMD (green circles) and a subject with late AMD (red circles). When assessed across the entire cohort of subjects, phagocytosis was found to be significantly reduced in monocytes isolated from subjects with both intermediate AMD and advanced disease (Figure 2C; One Way ANOVA, Tukeys *post-hoc*). Moreover, the reduction in phagocytosis was of a similar amount in all monocyte subtypes [~40% reduction in phagocytosis in those with AMD (iAMD or advanced AMD compared to controls)]. In view of the differences in age of the three populations of subjects, and the potential for age to influence phagocytic function, we performed an Analysis of Covariance (ANCOVA) to assess the effect of iAMD and late AMD on phagocytosis independent of age. For all monocyte types, iAMD and late AMD was associated with a lower level of phagocytosis compared to healthy controls (ANCOVA, data not shown).

The size of drusen is known to be an important risk factor for progression of disease (4). Therefore, we evaluated whether a higher burden of drusen was associated with lower levels of phagocytosis. We measured the area of the retina covered by drusen in the iAMD group within the central 5 mm of the fovea and correlated this with monocyte phagocytosis. As shown in Figure 2D there was no significant correlation between the level of phagocytosis and drusen area for any of the three monocyte subtypes evaluated.

**Figure 3:**
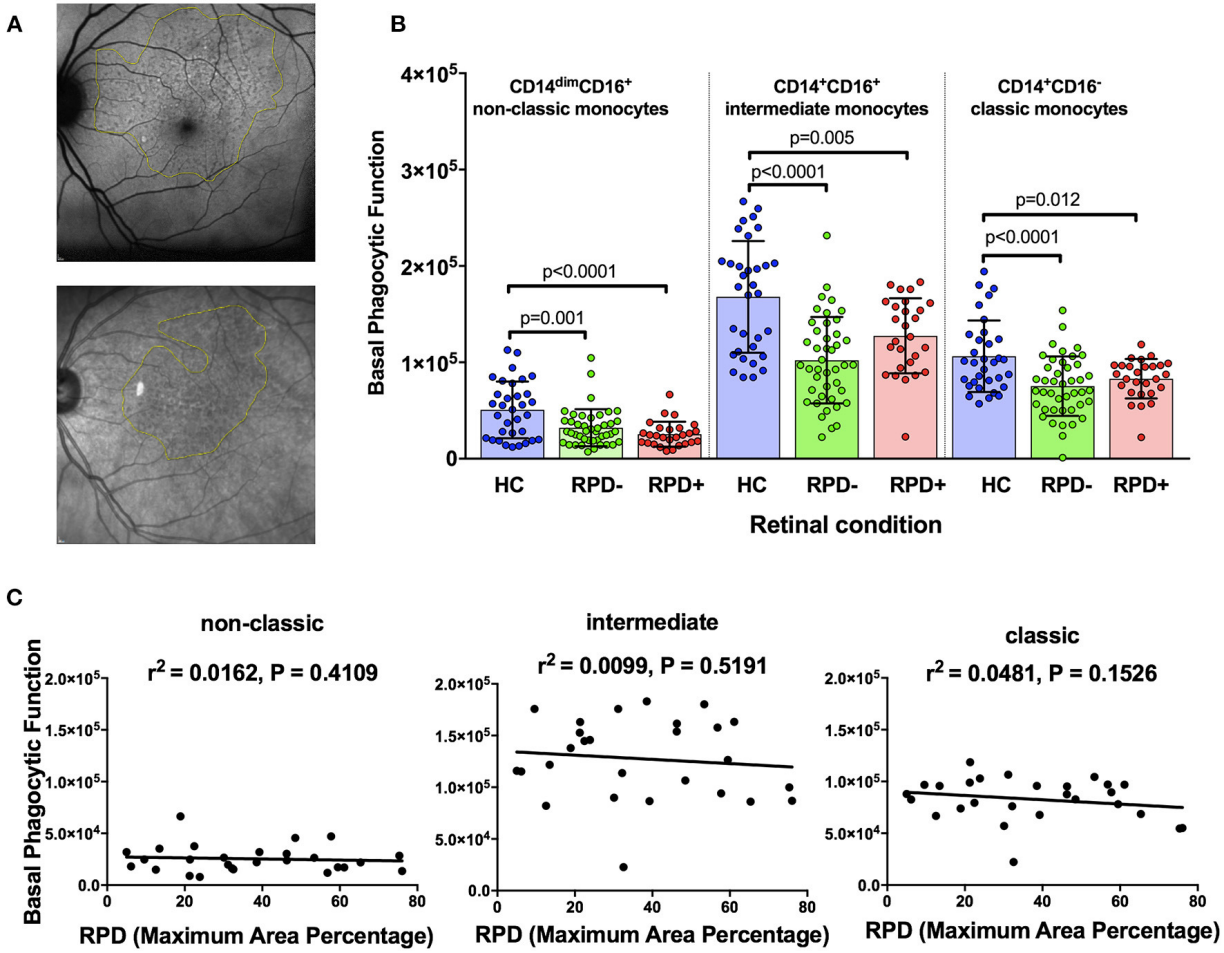
Phagocytic function in participants with and without reticular pseudodrusen **(A)** Fundus auto-fluorescence (FAF; top) and near Infrared reflectance (NIR; bottom) spectroscopy images showing reticular pseudodrusen (RPD) area outlined using Image J software. **(B)** Graph showing mean+standard deviation of monocyte phagocytosis in healthy controls (HC, *n* = 35), subjects with intermediate AMD without RPD (RPD^−^; *n* = 42) and intermediate AMD with RPD (RPD^+^; *n* = 18). *P*-values from One-way ANOVA analysis and Tukey's multiple comparisons tests are shown for comparison between specific groups. **(C)** Correlations of basal phagocytic function of three monocyte subsets with RPD area (*n* = 60). There was no significant correlation between phagocytosis and drusen area for any of the monocytes examined.

Reticular pseudodrusen (RPD) are a recently recognized deposit that has been associated with increased risk of disease progression and altered response to potential treatment such as with subthreshold nanosecond laser (7, 29). We examined whether phagocytic function was differentially affected in those with AMD and RPD (referred to here as RPD^+^) compared to those with conventional drusen only (RPD^−^). RPD were quantified by both near-infrared reflectance and fundus autofluorescence in HC (*n* = 35), RPD^−^ AMD (*n* = 42) and RPD^+^ AMD (*n* = 18) (Figure 3A). As shown in Figure 3B, all patients with intermediate AMD, irrespective of the presence of RPD showed reduced phagocytosis in all monocyte subsets. In addition, there was no significant correlation between the phagocytic function and RPD area for any of the monocyte types examined (Figure 3C). These effects were observed even when data was adjusted for age, gender or laser intervention (ANCOVA, data not shown).

### Glatiramer Acetate Restores AMD Monocyte Phagocytic Ability *in vitro*

**Figure 4:**
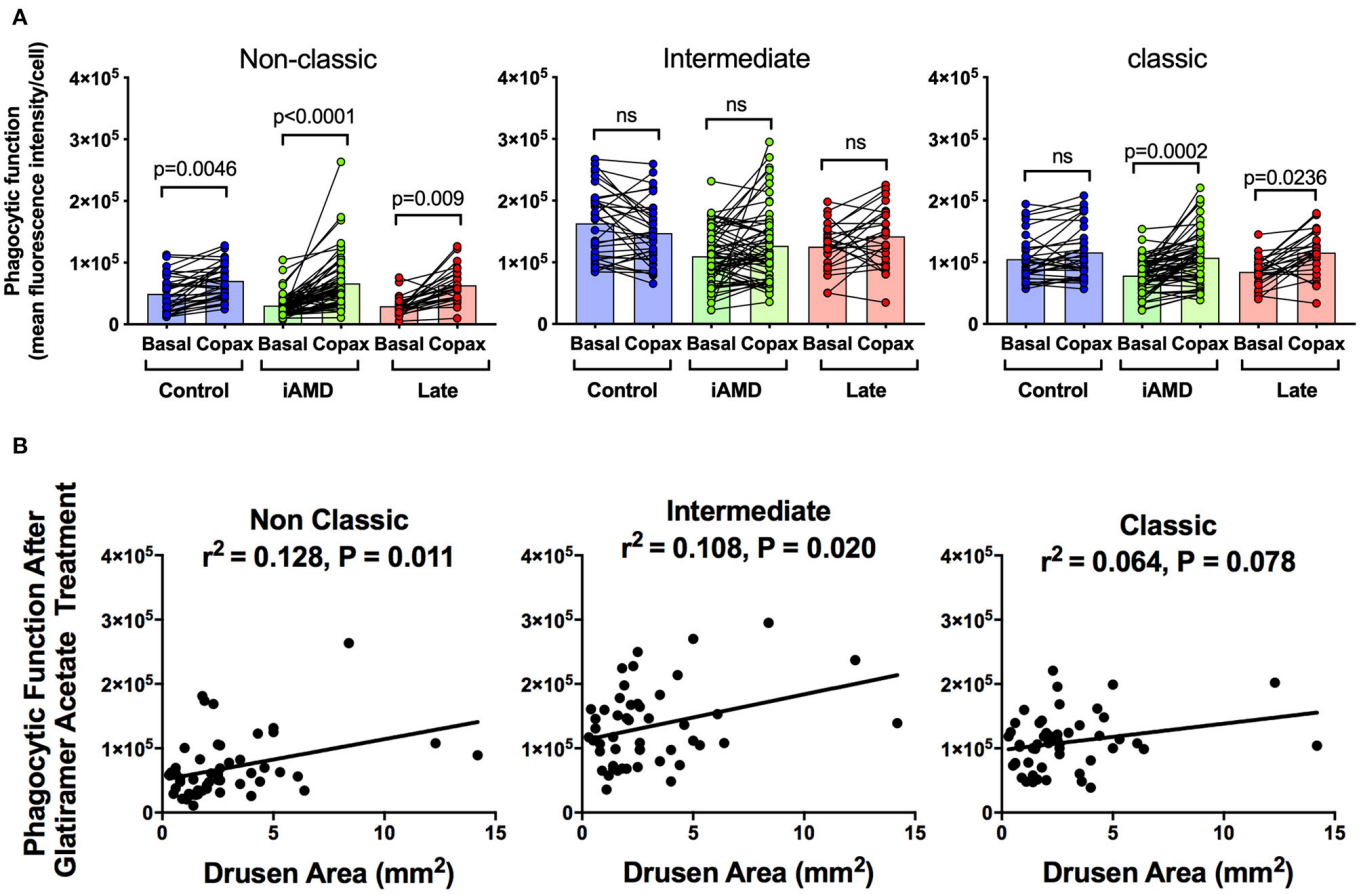
The effect of glatiramer acetate treatment on phagocytosis and its correlation with drusen size. **(A)** Graph of monocyte phagocytosis before and after 10 min treatment with glatiramer acetate 100 μg/mL for healthy controls, subjects with intermediate AMD (iAMD) and subjects with late AMD. *P*-values from a Two-way ANOVA analysis with *post-hoc* Tukey's-test are shown on top of each panel. **(B)** Correlations of drusen area with phagocytic function following glatiramer acetate treatment in subsets of monocytes. There was a significant correlation between drusen area and glatiramer acetate stimulated phagocytosis for non-classical and intermediate monocytes. ns: no significance.

Having shown a reduction in phagocytosis activity in all three monocytes in those with AMD, irrespective of stage of disease or monocyte type, we evaluated whether monocyte phagocytosis could be modified therapeutically. Glatiramer acetate (Copaxone®), is an approved compound used in the treatment of multiple sclerosis and is known to alter immune cell phagocytosis (27). We evaluated the potential of glatiramer acetate to modify monocytes by pre-treating monocytes isolated for all subjects for 10 min in 100 μg/mL glatiramer acetate at 37°C. Figure 4A shows phagocytic function of the three monocyte subtypes isolated from the healthy control, iAMD and advanced AMD subjects before and after application of glatiramer acetate (Copaxone). Glatiramer acetate enhanced phagocytosis of non-classical monocytes isolated from healthy control subjects by ~45% (*p* < 0.0046), whereas it had very little effect on phagocytosis by intermediate or classical monocytes. In contrast, phagocytosis by non-classic and classic monocytes was enhanced by treatment with glatiramer acetate in monocytes isolated from subjects with intermediate AMD (Figure 4A). These effects were observed even when data was adjusted for age, gender or laser intervention (ANCOVA, data not shown). Phagocytosis was also enhanced in non-classic and classic monocytes isolated from subjects with advanced AMD. Moreover, the enhancement in phagocytosis in both non-classic and intermediate monocytes was significantly correlated with drusen area (Figure 4B).

**Figure 5:**
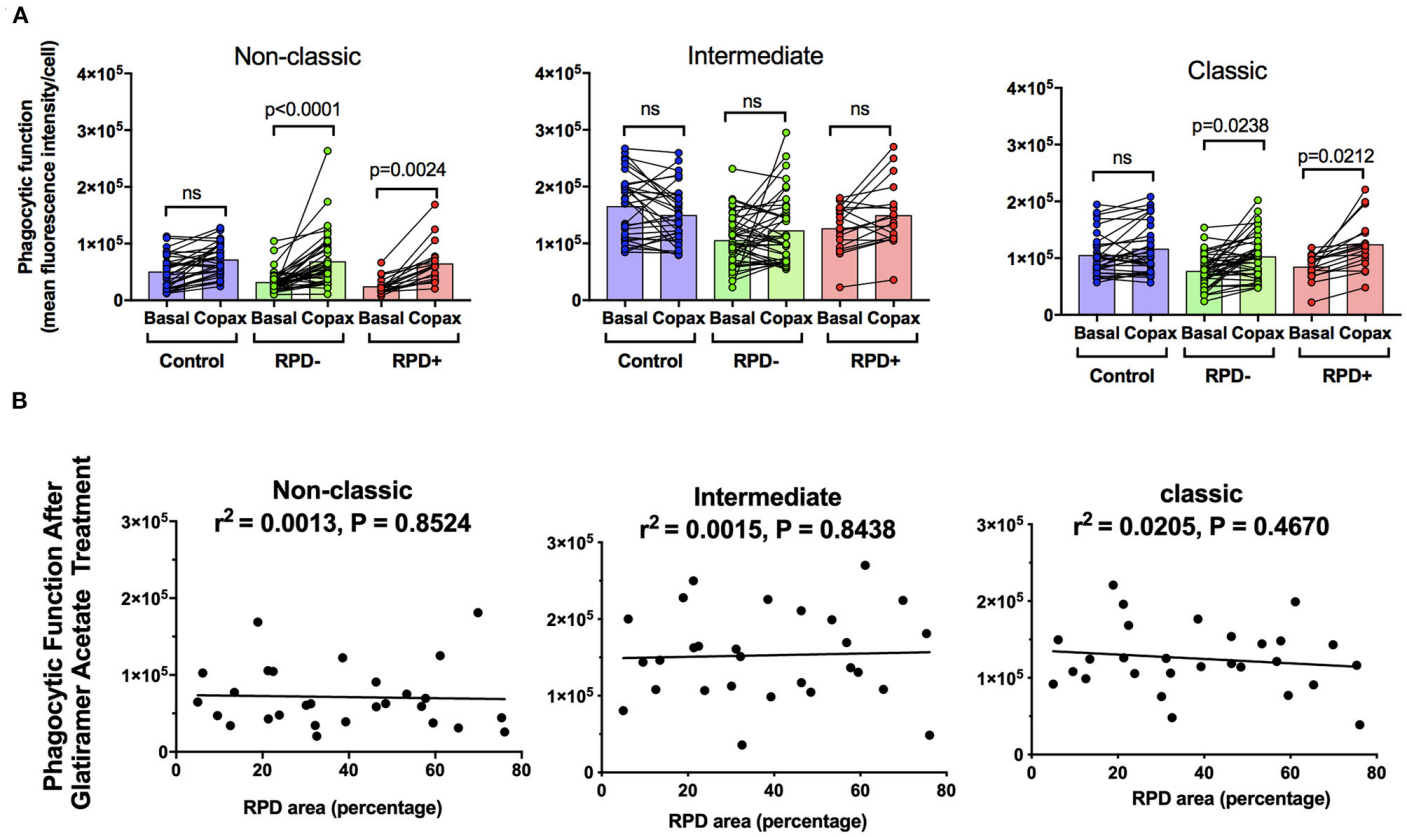
The effect of glatiramer acetate treatment on monocytes phagocytosis isolated from those with and without RPD and its correlation with RPD area. **(A)** Graph showing phagocytic function before and after 10-min application of glatiramer acetate in monocytes isolated from healthy controls, subjects with intermediate AMD without RPD (RPD^−^) and subjects with intermediate AMD with RPD (RPD^+^). *P*-values from a Two-way ANOVA with *post-hoc* Tukey's analysis analysis are shown on top of each panel. **(B)** Correlations of RPD area with phagocytic function following glatiramer acetate treatment in subsets of monocytes. There was no significant correlation between RPD area and glatiramer acetate stimulated phagocytosis. ns: no significance.

Figure 5 shows the effect of glatiramer acetate on monocyte phagocytosis isolated from subjects intermediate AMD with and without RPD. Glatiramer acetate enhanced phagocytosis of non-classic and classical monocytes isolated from subjects with intermediate AMD, irrespective of the presence of RPD (Figure 5A). These effects were observed even when data was adjusted for age, gender or laser intervention (ANCOVA, data not shown). However, there was no correlation between the effect of glatiramer acetate on phagocytosis and area of the retina covered by RPD (Figure 5B). A larger area of retina covered by RPD was not associated with a higher effect of glatiramer acetate on phagocytosis.

**Figure 6:**
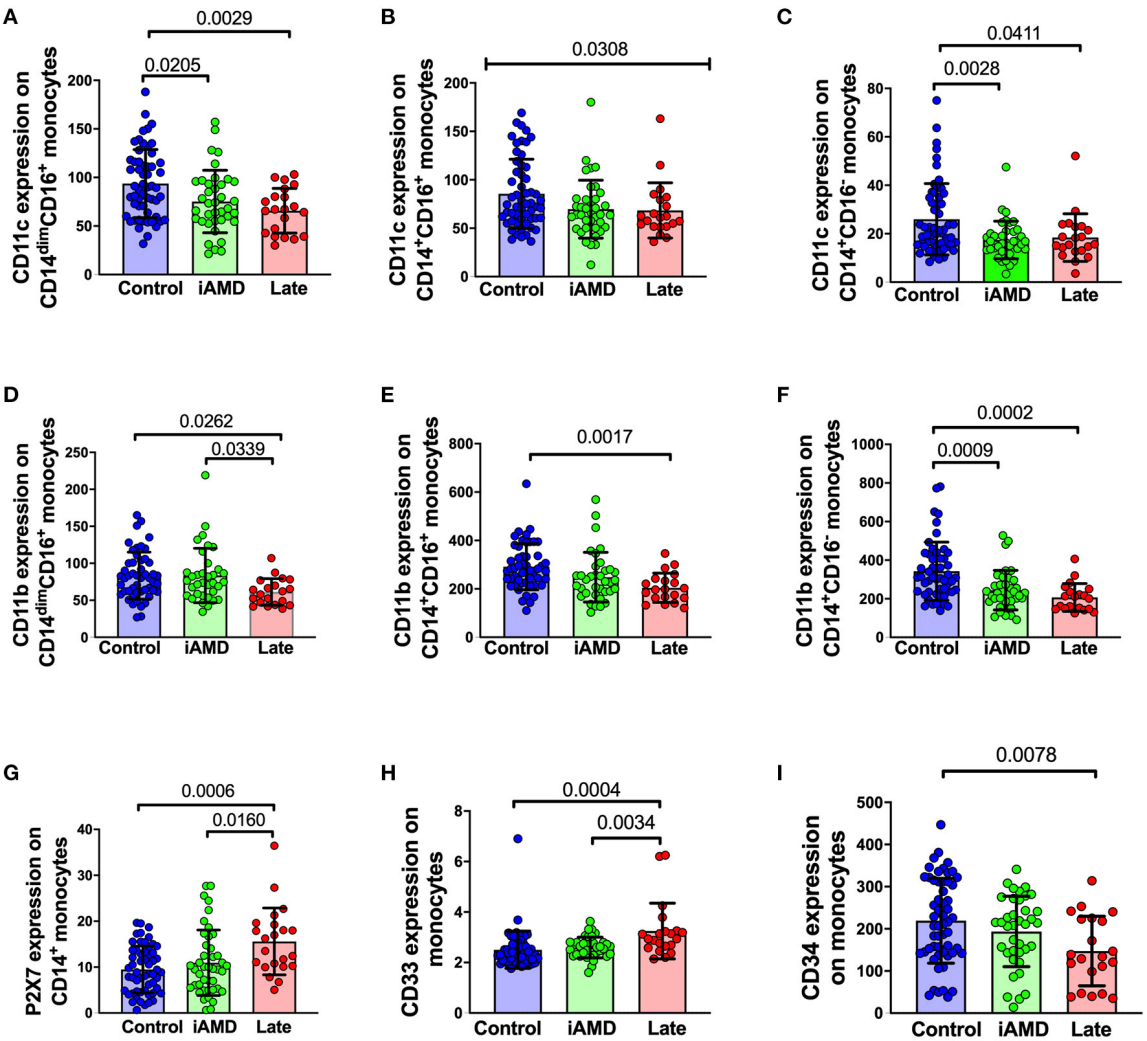
Surface expression of phagocytosis related molecules in leukocyte from HC, early and late AMD. Whole blood leukocytes were labeled with different surface markers in four-color flow cytometry panels. Samples were from healthy controls (*n* = 52), early AMD (*n* = 39) and late AMD (*n* = 22). Parameters that showed significant differences are shown. *P*-values from One-way ANOVA analysis and Tukey's multiple comparisons tests are shown for comparison between specific groups. In B, the *p*-value for the One-Way ANOVA across all three groups is shown.

Surface expression of phagocytosis-associated molecules in AMD and control participants Phagocytosis is known to depend on a range of surface receptors. To further explore the mechanisms underlying the defective phagocytosis found in AMD patients, we examined the surface expression of a number of receptors and molecules associated with innate phagocytosis on peripheral blood leukocytes. Integrins are important membrane receptors regulating leukocyte adhesion, migration, phagocytosis and many other activities (32). We investigated the expression of two well-known membrane receptors of this family: CD11b and CD11c. The integrins, CD11b and CD11c are known to form complexes with CD18 and are referred to as complement receptor 3 (CR3, CD11b/CD18) and complement receptor 4 (CR4, CD11c/CD18) respectively (33, 34). These receptors are known to mediate phagocytosis via mechanisms involving complement and/or antibody opsonization (32). In view of the known importance of the complement pathway in AMD, we reasoned that a reduction in these integrins may contribute to the reduced phagocytosis observed in patients. As shown in Figures 6A–F, the surface expression of both CD11c and CD11b across the three subsets of monocytes were all reduced. Besides integrins, we also examined some other surface molecules. P2X7 and CD33 are known phagocytosis-related biomarkers (25, 35). However, both molecules showed increased expression on the surface of monocytes (Figures 6G,H), especially when those with late disease were compared with healthy controls. Finally, we tested for CD34, a marker of haemopoietic stem cells was reduced in those with late AMD, implying that the capacity for differentiation of haemopoietic stem cells into monocytes was reduced in those with late AMD (Figure 6I).

### Correlations of Leukocyte Surface Biomarkers With Drusen or RPD

**Figure 7:**
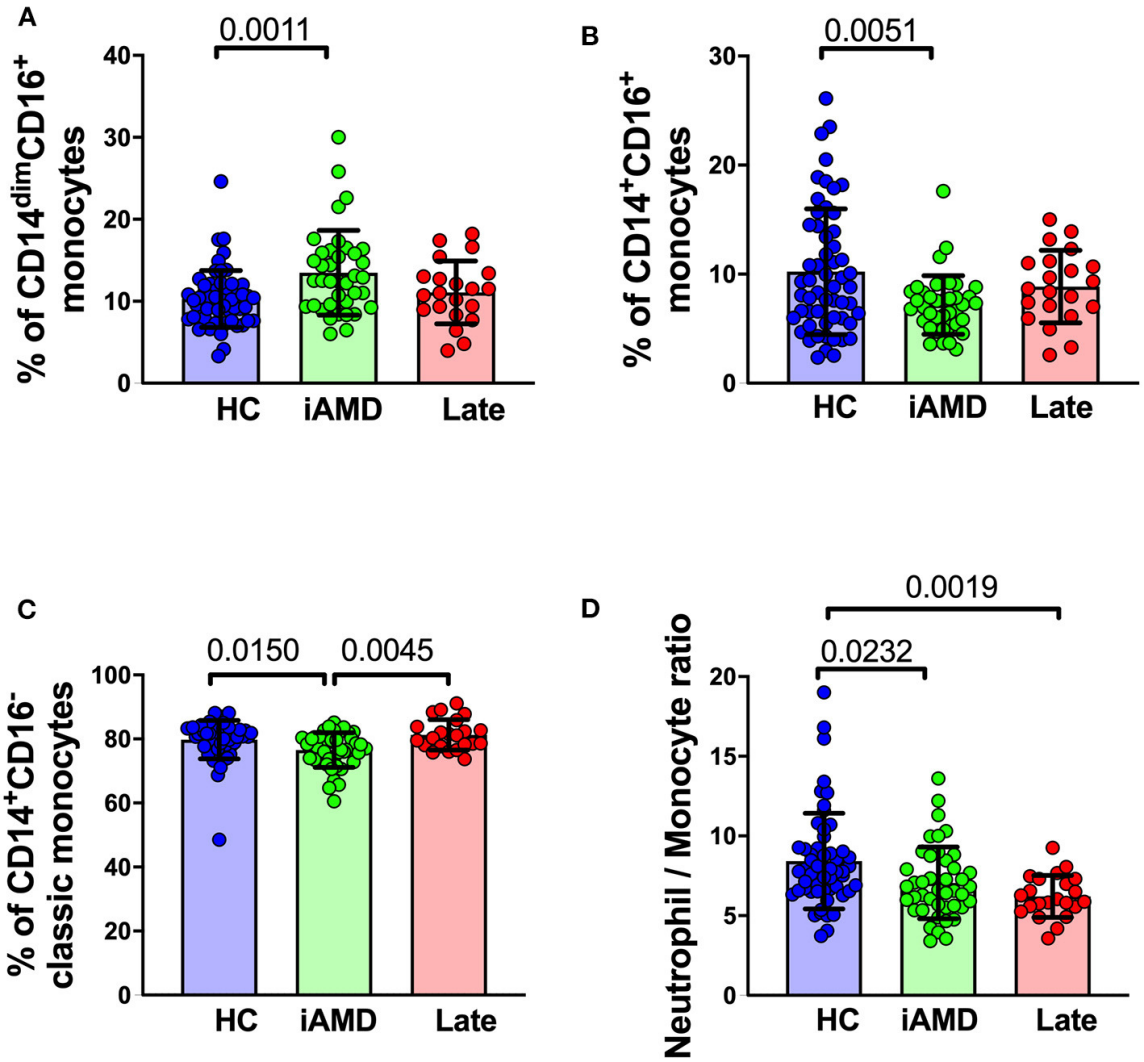
Changes in proportions of monocyte subtypes in healthy control, early and late AMD subjects Graph of the mean ± SD proportion of **(A)** non-classical monocytes (CD14^−^CD16^+^), **(B)** intermediate monocytes (CD14^+^CD16^+^), **(C)** classic monocytes (CD14^+^CD16^−^) and **(D)** and ratio of neutrophils to monocytes. Samples were from healthy controls (*n* = 35), intermediate AMD (*n* = 42) and late AMD (*n* = 30). Parameters that showed significant differences are shown. *P*-values from One-way ANOVA analysis and Tukey's multiple comparisons tests are shown for comparison between specific groups.

It is well-known that individuals with larger drusen have a greater risk of progression to vision threatening stages (4). We evaluated whether there were differences in monocyte proportions in those with a larger overall load of drusen compared with those with a smaller drusen load. Figure 7 shows changes in the proportion of the different subsets of monocytes in healthy control subjects, those with intermediate AMD and those with late AMD. Overall, classic monocytes (CD14^+^CD16^−^) comprise ~80% of the entire monocyte population, and intermediate and non-classical monocytes represent only ~10% of the total monocyte population. In those with intermediate AMD there as an increase in the proportion of non-classical monocytes (CD14^dim^, CD16^+^; One-way ANOVA, *p* = 0.0011) and a corresponding decrease in the proportion of classical (CD14^+^CD16^−^; One Way ANOVA, *p* = 0.015) and intermediate monocytes (CD14^+^CD16^+^; One Way ANOVA, *p* = 0.005). In contrast, the proportion of monocytes in late AMD was no different from health controls for any monocyte type. In agreement with these findings, the ratio of neutrophils to monocytes was reduced in those with intermediate and late AMD (Figure 7D; One Way ANOVA, *p* = 0.023). The proportions of other cell types, including neutrophils and lymphocytes were unchanged (data not shown). These significant differences were observed even when considered independently of age and gender (ANCOVA).

### Phagocytosis-Associated Leukocyte Surface Molecules Have the Potential Prognostic Values for AMD Progression

**Figure 8:**
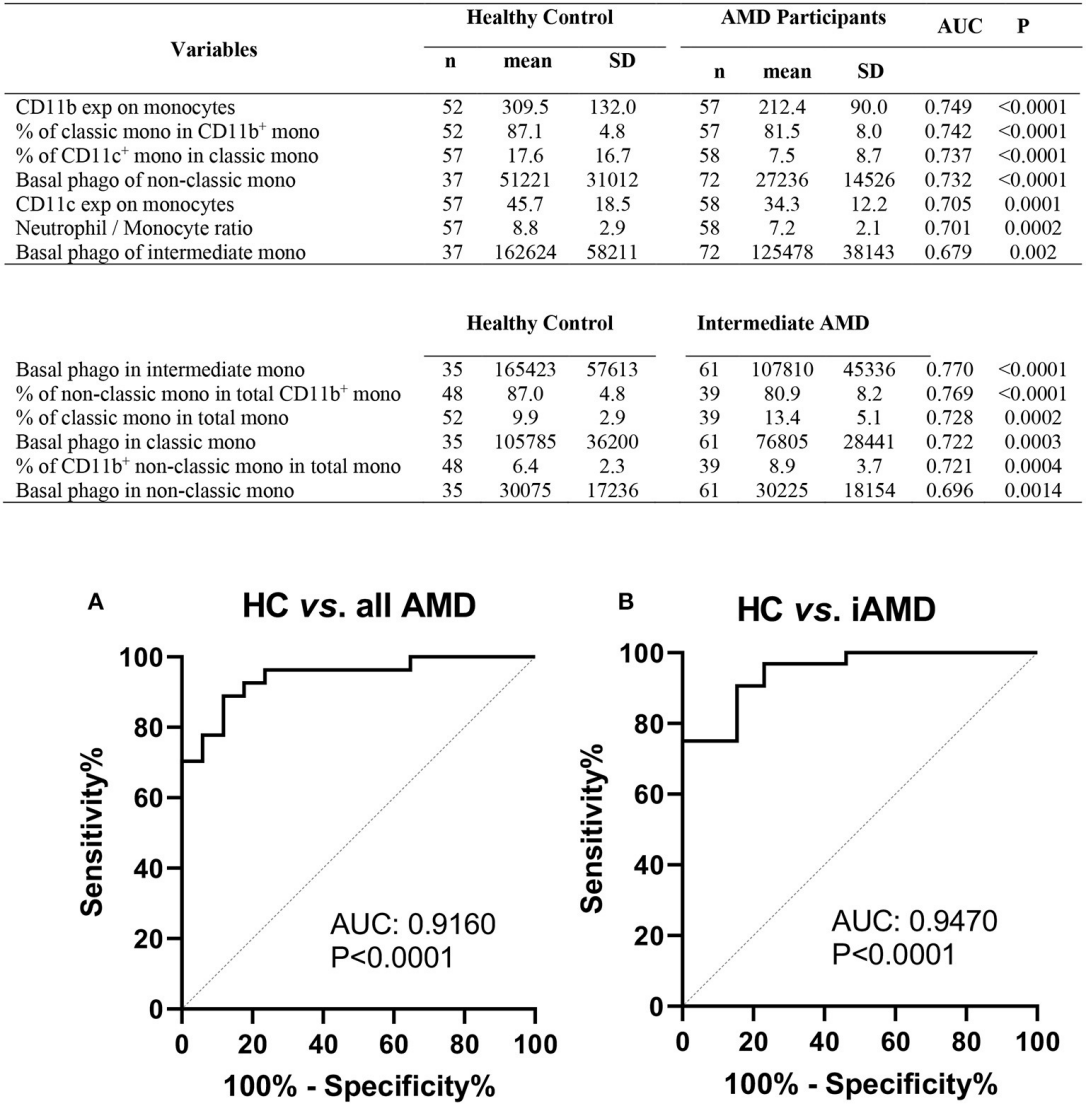
Phagocytic function and leukocyte biomarkers with potential diagnosis values for AMD. Top potential variables for diagnosis of AMD are shown in the table. AUC, area under curve of Receiver Operating Characteristic (ROC); Mono, monocytes; exp, surface expression; SD, standard deviation; phago, phagocytic function. Binary logistic regression was performed among selected multiple variables to create predicted probabilities for ROC analysis. ROC curves distinguishing between **(A)** healthy controls (HC) and all AMD (intermediate, GA and CNV) and **(B)** HC and intermediate AMD are shown. *P*-values are for asymptotic significance calculated by SPSS v24.

**Figure 9:**
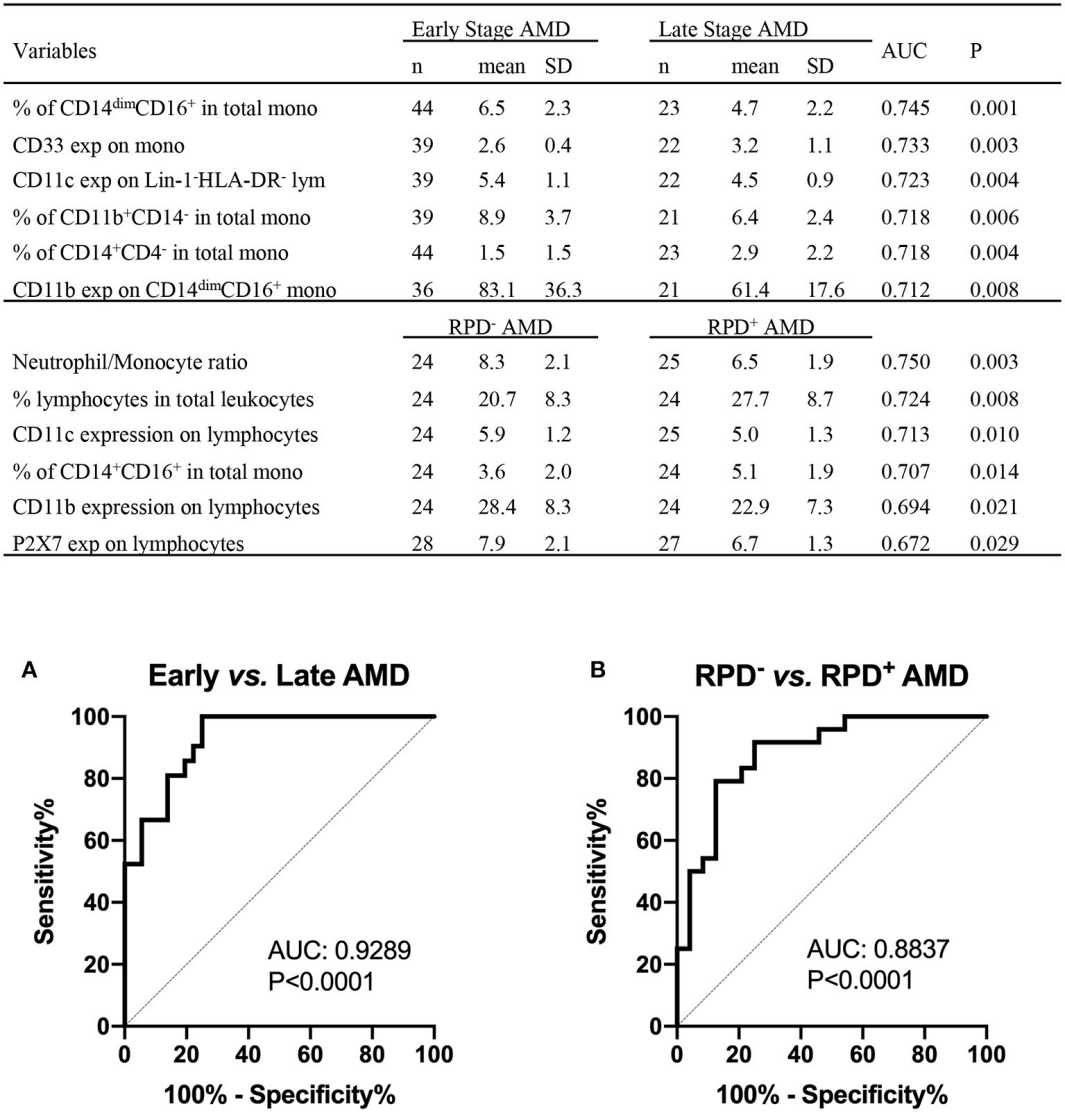
Leukocyte proportion and surface biomarkers with potential prognostic values for AMD. Top 6 potential variables for prognosis of AMD are shown in the table. AUC, area under curve of Receiver Operating Characteristic (ROC); Mono, monocytes; exp, surface expression; SD, standard deviation. Binary logistic regression was performed among multiple parameters to create predicted probabilities for ROC analysis. ROC curves distinguishing between **(A)** early stages (intermediate) and late stages of AMD (GA and CNV) and between **(B)** RPD^−^ and RPD^+^ AMD patients are shown. *P*-values are for asymptotic significance calculated by SPSS v24.

The results shown above suggest that monocyte function is altered in those with intermediate or late AMD. Although the different stages of AMD are readily diagnosed using ocular imaging, we evaluated whether leucocyte factors could be used to differentiate the different forms of disease. By comparing healthy controls and AMD participants using receiver operating characteristic (ROC) analysis, we identified several leukocyte surface variables which may have potential prognostic value identifying those with AMD (Figure 8), including phagocytic function of non-classic and intermediate monocytes and CD11b and CD11c expression by monocytes. Further comparison between healthy control and those with intermediate AMD indicated that phagocytic function of all monocyte classes in particular was a useful discriminator of those with intermediate AMD. Figure 9 shows receiver operating characteristic (ROC) analysis for those with intermediate AMD compared late AMD and demonstrates that with a combination of the top six selected variables (Figure 9), a theoretical 92.9% accuracy can be achieved that distinguishes early stage from late AMD. We took a similar approach to compare RPD^−^ and RPD^+^ AMD. With a combination of top six selected biomarkers, an 88.4% accuracy could be achieved in distinguishing AMD patients who also had RPD from those without RPD^−^ (Figure 9B). Notably, factors relating to lymphocytes including CD11b, CD11c, and P2X7 expression by lymphocytes as well as the proportion of lymphocytes were major distinguishing factors that separated those with RPD from those without RPD. It is worth noting that none of the leukocyte surface biomarkers were associated with age or sex of participants. These results highlight that there is a different leucocyte profile in the various types of AMD (i.e., early compared to late disease; presence or absence of RPD).

## Discussion

The main findings of this study were that both intermediate and late AMD were associated with a reduction in monocyte phagocytosis that could be ameliorated in part by application of glatiramer acetate. In addition, the presence of RPD had no additional effect on monocyte phagocytosis compared to that observed in those without RPD. These results highlight a novel systemic change in monocyte function in those with AMD that may reflect a novel mechanism of disease and could have the potential as a novel therapeutic target.

Our results showing that phagocytosis was reduced in all monocyte subtypes in patients with intermediate and late AMD, highlights that systemic monocyte function may be important in the development of AMD. Although, previous studies have shown that monocytes are altered in one late form of AMD—neovascular AMD (19, 36–41), no information is currently available about potential functional changes that occur in monocytes at an earlier stage of disease. Anomalies in the innate immune system have been implicated in the development and progression of AMD. Migration of mononuclear phagocytes into the subretinal space has been implicated in the changes that occur during the early stages of AMD. Notably, mononuclear phagocytes that includes peripheral blood monocytes, macrophages and retinal microglia, accumulate within and around drusen, RPD, and also in zones of atrophy (11). In addition, mononuclear phagocytes accumulate within the subretinal space with age in animal models with features of early stages of AMD and can influence both photoreceptor and RPE integrity. Indeed, reduced mononuclear phagocyte phagocytosis has been previously associated with enhanced neuroinflammation and accelerated photoreceptor loss (42). When taken together with these previous observations, we propose that a reduction in monocyte phagocytosis observed in subjects with AMD could be important in for the development of deposits, and potentially contribute to the ongoing pathological changes that occur in the posterior eye during AMD. However, additional studies are required to identify a causal link between monocyte phagocytosis and development and/or progression of AMD.

A number of previous studies have highlighted anomalies in monocyte function in those with neovascular AMD. Notably, the proportions as well as chemokine receptor expression of monocytes is altered in subjects with neovascular AMD (40, 43–45) and monocytes isolated from those with neovascular AMD secrete greater levels of cytokines including VEGF (46). Our findings showing reduced expression of CD11b in all monocyte subtypes isolated from those with late AMD is in agreement with these previous studies. CD11b is an integrin that combines with CD18 to form the complement receptor 3 complex, an important mediator of phagocytosis of complement- coated particles. CD11c, another member of the integrin family, is also thought to be important for mediating phagocytosis, via a mechanism involving binding to iC3b. Our results showing reduced monocyte phagocytosis in combination with reduced expression of CD11b and CD11c suggest that complement mediated phagocytic mechanisms may be perturbed in those with neovascular AMD.

Anomalies in monocyte function have been implicated in a number of diseases known to be associated with anomalies in innate immune signaling including multiple sclerosis, Alzheimer's disease and Parkinson's disease (28, 47). Defective myelin phagocytosis by monocytes has been implicated in increased inflammation in those with multiple sclerosis (48). Similarly, defective monocyte phagocytosis is associated with elevated α-synuclein, and increased inflammation in those with Parkinson's disease (47, 49, 50). Our previous study demonstrated that individuals with mild cognitive impairment or Alzheimer's disease associated with a high Aβ burden, had higher levels of monocyte phagocytosis compared to healthy controls (28). The results of this study confirm that assaying peripheral blood monocyte function can provide important information about the role of the innate immune system in disease.

The underlying mechanisms leading to reduced monocyte phagocytosis remain to be determined. Age has been associated with reduced monocyte phagocytosis in one study (51), and our experimental groups did differ in age between the healthy controls and the late AMD cases. However, we found no significant correlation between phagocytosis and age in our cohort and when data was adjusted for age and gender there was no impact on experimental outcomes- phagocytosis was reduced in subjects with iAMD and Late AMD. Moreover, our observation that AMD was associated with reduced phagocytosis is unlikely to be due to aging because the mean age of control subjects was older than our subjects with intermediate AMD.

It has been reported that monocytes isolated from those with late AMD have a distinct transcriptome profile (19) as well as modified DNA methylation compared to monocytes isolated from health aged matched controls (37). Our previous work has implicated the scavenger receptor, P2X7 in AMD (21, 22). The P2X7 receptor has a tight molecular association with non-muscle myosin heavy chain IIA (NMMHC-IIA) in monocytes (24) and this complex regulates phagocytosis of non-opsonized beads, live and dead bacteria, as well as apoptotic cells (25). Our previous report examining P2X7null mice, shows that rate of monocyte phagocytosis in peripheral blood monocytes reduces with age, and is associated with gradual thickening of Bruch's membrane, a critical change in the development of AMD (22). In addition, we have found that a rare haplotype of P2X7 G150R together with P2X4 Y315C leads to loss of innate phagocytosis and confers increased risk of late AMD (21). However, in view of the large number of participants showing reduced monocyte phagocytosis in this study, there are likely to be additional scavenger receptor types and cellular processes involved. More work is needed, to determine whether additional scavenger receptors, or cellular processes affecting monocyte phagocytosis are affected in those with AMD.

Recently, the importance of a distinct deposit called RPD has emerged (5–7). RPD form within the subretinal space between photoreceptor outer-segments and the RPE and have a distinct composition compared to conventional drusen (7, 52). The underlying mechanisms leading to the formation of RPD compared to conventional drusen are not well-understood (7). We evaluated whether patients with RPD showed altered monocyte function compared to those with conventional drusen. Our results indicate that phagocytosis by all subsets of monocytes was uniformly reduced in all participants with AMD, irrespective of the presence of RPD suggesting that defective phagocytosis is similarly abnormal in those with or without RPD.

Treatment with glatiramer acetate was found to ameliorate the changes in phagocytosis associated with the intermediate and late AMD. These positive effects were observed in non-classical and classical monocytes isolated from patients with or without RPD. Moreover, glatiramer acetate had a greater effect on monocyte phagocytosis isolated from patients with a greater drusen load. These results are in agreement with our previous study showing glatiramer acetate enhancement of monocyte phagocytosis in individuals with Alzheimer's disease or mild cognitive impairment associated with high levels of Aβ (28). Our previous study showed that the higher the level of Aβ, the greater the effect that glatiramer acetate had on monocyte phagocytosis. Aβ is known to be enriched in drusen and it is therefore possible that similar biological mechanisms underpin the enhanced phagocytosis induced by glatiramer acetate in those with AMD. Glatiramer acetate is approved for the treatment of multiple sclerosis and although its mode of action is not well-understood, it is thought to target T cell as well as monocyte function (26–28). The mechanism by which glatiramer acetate changes monocyte phagocytosis is, however, not clear. Our previous work has shown that glatiramer acetate interacts rapidly with the cell membrane of monocytes, perhaps potentiating the recognition of particles by scavenger receptors (28). In addition, a previous small study has demonstrated resolution of drusen in those with AMD treated for 12 weeks with subcutaneous glatiramer acetate (53, 54). More work is needed, however, to determine whether *in viv*o treatment with glatiramer reduces the development or progression of AMD.

Our results indicate that in those with AMD, glatiramer acetate selectively enhances phagocytosis of non-classical and classical monocytes but has little effect on intermediate monocytes. The selective effect of glatiramer acetate on specific monocyte subtypes is consistent with our previous study that showed a preferential enhancement of phagocytosis by non-classical and classic monocytes in healthy control subjects, or those with a high burden of Aβ (28). It is possible that glatiramer acetate preferentially enhances phagocytosis in cell types that show low basal phagocytosis such as non-classic or classic monocytes. There may be a limitation in the ability to enhance phagocytosis, once a threshold level has been attained.

The results of this study should be viewed in the context of a number of limitations. This study was a cross-sectional, *in vitro* laboratory study and, the findings are yet to be validated in a longitudinal manner. Importantly, the association between AMD and phagocytosis observed in this study requires further investigation to determine whether there is a direct causal link between monocyte phagocytosis and development or progression of disease. Secondly, there are a number of co-morbidities that are known to alter innate immune cell function and that may have influenced our results, including aging, sex, and lifestyle factors such as smoking. Although our analysis failed to detect any effect of sex or age on phagocytic function, other lifestyle factors were not accounted for in our analysis. Finally, our results demonstrating the effect of glatiramer acetate on monocyte phagocytosis was performed *in vitro*. A follow-up study is required to determine whether glatiramer acetate influences monocyte phagocytosis *in vivo* and whether this can change disease progression.

In conclusion, this study has shown an identifiable and measurable systemic defect in innate immune cell function in a cohort of AMD cases that cover the spectrum of disease severity. Moreover, *in vitro* treatment with glatiramer acetate ameliorated the reduced phagocytic function in AMD participants with intermediate and late AMD. Importantly, our results show that glatiramer acetate was able to restore monocyte phagocytosis in the most common subtypes of monocytes, non-classical and classical monocytes, to a level similar to healthy controls, suggesting a potential novel intervention for the earlier stages of AMD, before there are vision threatening complications of atrophy or neovascularization. Further work is needed to elucidate the contribution that reduced phagocytosis has to the development and progression of AMD, and in particular to evaluate whether glatiramer acetate has potential as a therapeutic agent for reducing drusen and/or reticular pseudodrusen burden.

## Data Availability Statement

The raw data supporting the conclusions of this article will be made available by the authors, without undue reservation.

## Ethics Statement

The studies involving human participants were reviewed and approved by The Royal Victorian Eye and Ear Human Ethics committee. The patients/participants provided their written informed consent to participate in this study.

## Author Contributions

BG, RG, and EF: contributed equally to study design, data interpretation, and writing. XH, PA, CD, EC, KV, AO, CF, T-HL, YL, and AH: data collection and data analysis. CM and JW: data interpretation and writing. All authors: contributed to the article and approved the submitted version.

## Conflict of Interest

The authors declare that the research was conducted in the absence of any commercial or financial relationships that could be construed as a potential conflict of interest.
